# Foraging ecology of the amphibious mudskipper *Periophthalmus chrysospilos* (Gobiiformes: Gobiidae)

**DOI:** 10.7717/peerj.12582

**Published:** 2021-12-07

**Authors:** Quang Minh Dinh, Ton Huu Duc Nguyen, Tran Thi Huyen Lam, Tien Thi Kieu Nguyen, Giang Van Tran, Zeehan Jaafar

**Affiliations:** 1 Department of Biology, School of Education, Can Tho University, Can Tho, Vietnam; 2 Department of Molecular Biotechnology, Biotechnology Research and Development Institute, Can Tho Univeristy, Can Tho, Vietnam; 3 Department of Pharmacy, Cuu Long University, Vinh Long, Vietnam; 4 Department of Biology, An Khanh High School, Can Tho, Vietnam; 5 Department of Zoology, Faculty of Biology, Hue Univeristy of Education, Thu Thien Hue, Vietnam; 6 Department of Biological Sciences, National University of Singapore, Singapore

**Keywords:** Carnivorous, Specific feeder, Food composition, Mudskipper

## Abstract

The food composition and feeding ecology of fishes living in the intertidal zone play an essential role in understanding the energetic connectivity between terrestrial and aquatic systems. *Periophthalmus chrysospilos* is an amphibious fish species occurring in the intertidal zone, but data on its diet and foraging ecology is still poorly known. This study on *Ps. chrysospilos* was carried out from April 2020 to March 2021 at four sites within the Mekong Delta estuary to define the influence of spatio-temporal factors on the diet of this species. The diet composition and relative gut lengths (RGLs) of *Ps. chrysospilos* were analysed in relation to four parameters—sex, size, site, and season. A total of 1,031 individuals were collected, and their digestive tract lengths were used to calculate the RGL. The digestive tracts of only 546 individuals were with food items (approximately 1:1 of empty *vs* full digestive tract) and were subsequently used for further analyses. The ranges in total length and weight in both adult and juvenile individuals were 3.4–10.6 cm and 0.38–14.13 g, respectively. The RGL values varied with season, fish size and site, but was always lower than 1, indicating a predominantly carnivorous diet. The variability of food items found within the digestive tracts demonstrated its adaptability in pursuing prey items within the limits of the littoral zone, and its importance as a conduit of terrestrial-marine connectivity. This species is characterised as an opportunistic mesopredator feeding primarily on *Acetes* spp., *Uca* spp., *Dolichoderus* sp., and rarely on Polychaeta and Actinopterygii. Other items found within the digestive tract are Mollusca, and detritus. The diet composition of *Ps. chrysospilos* did not vary with season and size, but changed with sex and site parameters. *Uca* spp. contributed to the sexual variation in dietary component, whereas Mollusca, *Uca* spp., *Dolichoderus* sp. and detritus, were drivers for spatial variation in the dietary component. The research provides fundamental information on diet composition and feeding strategy, as well as contributes towards knowledge on foraging ecology and resource use by intertidal animal communities.

## Introduction

Fishes, occupying all trophic levels within aquatic environments, are integral components to elucidate aquatic trophic ecology (Wootton, 1996; Blaber, 2000). In intertidal systems where terrestrial elements factor significantly in trophic ecology, studies traditionally focus on the impact of the environment on the foraging strategy of fishes (Ravi, 2013; Tran, Hoang & Dinh, 2019; Dinh et al., 2020b). During the ebb tide, the landscape of intertidal areas changes drastically. Mudskippers are some of the few fishes that have adapted to aerial exposure, and remain on these exposed littoral areas where they actively forage (Murdy & Jaafar, 2017). There are forty-three species of mudskippers, with varying sensitivities to aerial exposure and foraging behaviour (Jaafar & Murdy, 2017). Some species, such as those from the genus *Boleophthalmus*, are less tolerant to desiccation, stay close to the waterline, and graze the substrate surface for diatoms (Ravi, 2013; Dinh, 2015). Others, such as species of *Periophthalmodon*, are carnivorous and are known to traverse upriver to areas unaffected by tidal cycles (Dinh, 2018a, 2018b; Dinh, Tran & Nguyen, 2018; Dinh et al., 2020b).

Of the ten genera of mudskippers, the genus *Periophthalmus* is most speciose with 19 species and considered to be most tolerant to aerial exposure (Jaafar & Murdy, 2017; Dinh et al., 2021a). These fishes are extremely active during the ebb tide. Individuals are observed to forage throughout the mangrove zones, defend their territories, pursue mates, and maintain burrows; the latter oft in pairs (Polgar & Crosa, 2009; Murdy & Jaafar, 2017). *Periophthalmus* (*‘Ps*.’ hereafter) species are omnivorous and opportunistic foragers, and these are reflected in their morphology. Their teeth are typically caniniform, thick basally and recurved at the tips (Sponder & Lauder, 1981), while their gill rakers are short, knob-like projections that are widely separated (Mazlan, Masitah & Mahani, 2006). However, plant material is often found in the digestive tracts although there is still no consensus on its role in their diet. Some studies for example, report it as a principal diet component (Bob-Manuel, 2011; Udoh et al., 2013), while others consider it incidental intake (Clayton, 2017) or an alternate food source during winter months (Mhaisen & Al-Maliki, 1996). Species of *Periophthalmus* are known to be visual (Clayton, 1993; Kutschera, Burghagen & Ewert, 2008) and tactile hunters (Colombini et al., 1996; Somerfield, Gee & Aryuthaka, 1998), possessing well-developed olfactory epithelia (Kuciel, Żuwała & Jakubowski, 2011; Kim, Yun & Park, 2019) important receptors of olfactory cues during foraging bouts.

The prominence of these mesopredators within the intertidal zone, makes species of *Periophthalmus* excellent candidates to understand the energetic connectivity between terrestrial and aquatic systems. Yet, current information on the effects of spatio-temporal factors on the diet and foraging ecology of these fishes remain scarce (Ravi, 2013; Tran, Hoang & Dinh, 2019; Dinh et al., 2020b). We study the diet composition of an obligate intertidal species, *Periophthalmus chrysospilos* Bleeker 1853 (Gobiidae: Oxudercinae). Unlike all other species within this genus, *Ps*. *chrysospilos* forage in small groups of 20–40 individuals along the ebbing waterline (Milward, 1974; Polgar & Crosa, 2009). Through the analyses of food items within digestive tracts, we aim to define the food composition and investigate if the diet of this species changes with size, sex, site, and season. Our study on the diet composition of this species contributes towards knowledge on foraging ecology and resource use by intertidal animal communities.

## Materials and Methods

### Sampling sites

**Figure 1:**
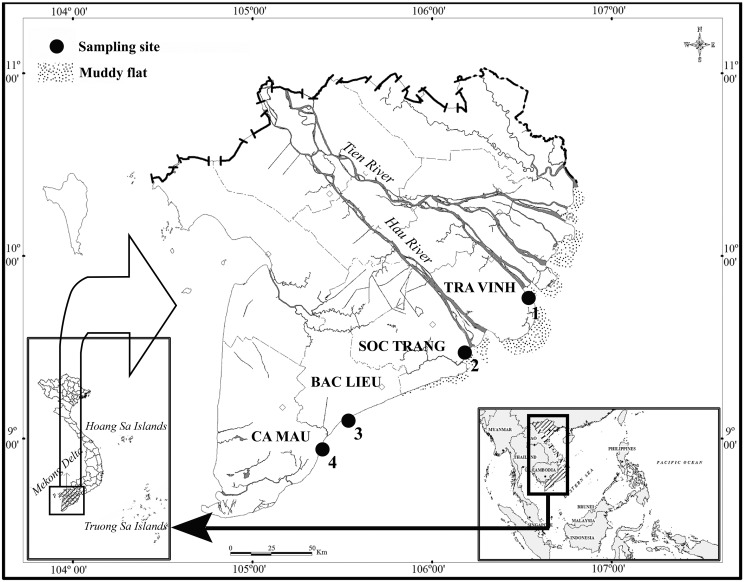
Four sites of this study indicated within the map in the Mekong Delta. Source credit: Dinh (2017) © John Wiley and Sons.

Specimens were collected from April 2020 to March 2021 at four locations along the Hau river estuary in Vietnam (Fig. 1): Duyen Hai-Tra Vinh (DHTV; 9°41′18.6″N 106°30′35.8″E), Tran De-Soc Trang (TDST; 9°29′26.8″N 106°11′58.5″E), Dong Hai-Bac Lieu (DHBL; 9°06′03.2″N 105°29′49.1″E); and Dam Doi-Ca Mau (DDCM; 8°58′17.5″N 105°22′51.8″E). There is little fluctuation in temperature at the sites between wet (June–December) and dry (January–May) seasons; the temperature remains at approximately 27 °C. Conversely, precipitation measures varied significantly—a monthly average of 20 mm in the dry season and 400 mm in the wet season (Le et al., 2006). The pH was 7.6–7.9 and varied with sites but not seasons, whereas the salinity was 12.3–23.5% and varied with season but not site (Dinh et al., 2021b).

Vegetation typical at these sites include *Acanthus ebracteatus* Vahl., *Avicennia marina* (Forssk.) Vierh., *Bruguiera gymnorrhiza* (L.), *Nypa fruticans* Wurmb., Savigny *Sonneratia caseolaris* (L.) A. Engl., and *Rhizophora apiculata* Blume. The dominant plant species at DHTV, TDST and DHBL were *Sonneratia caseolaris*, *Avicennia marina* and *Bruguiera gymnorrhiza* (L.) Savigny., respectively. At DDCM, *Avicennia marina* and *Bruguiera gymnorrhiza* (L.) Savigny. were equally dominant (Dinh et al., 2021b).

### Sample collection

Fishes were randomly collected ∼4 hours during the ebb tide by hand in an area of 120 square metres (6 m × 15 m) on the mudflat at night for five consecutive days per month. Fishes were identified following Murdy & Jaafar (2017) and sexed. The genital papillae of females are bulbous, pinkish, and equally broad at the base and tip, whereas those for males are slender and whitish, broad at the base and tapers towards the tip (Dinh et al., 2020a). Fishes captured were immediately euthanised in a solution of tricaine methanesulfonate (MS222) before being transferred into a solution of 5% buffered formalin and transported to the laboratory. In the laboratory, specimens were soaked in water for 2 hours before analyses. The total length (TL, nearest to 0.1 cm) and weight (W, nearest to 0.01 g) of each specimen was recorded before dissection and analyses of content of the digestive tract. The length at first maturity (*L*_*m*_) was used to divide the fishes into two groups: immature group if TL < *L*_*m*_ and mature group if TL ≥ *L*_*m*_. Individual of females and males from DHTV with total lengths of 7.3 cm and 6.9 cm or below were considered immature. *L*_*m*_ values of females and males from TDST were 6.4 cm and 6.6 cm; 7.0 cm and 6.2 cm from DHBL; and 6.8 cm and 8.6 cm from DDCM (Q. M. Dinh, 2021, Unpublished data).

The use of fishes in the present study was assessed and approved by The Council for Science and Education, School of Education, Can Tho University (Animal Welfare Assessment number: BQ2020-03/KSP). Altogether, 1,031 specimens were used for RGL and 546 specimens for diet composition analyses.

### Relative gut length

The digestive tract of each specimen was carefully removed and measured (nearest 0.1 cm) to obtain the relative gut length (RGL = total length of the gut/the total length of fish). The association of the length of digestive tract to the length of the fish has been used as an indicator for feeding guild (see Al-Hussaini (1947), Kapoor, Smit & Verighina (1976), Drewe et al. (2004)). Typically, RGL value greater than 3 signifies that the species is herbivorous, RGL value between 1 and 3 signifies omnivorous fishes, and RGL value below 1 signifies carnivorous fishes.

### Diet composition

Contents of each digestive tract were analysed under the stereomicroscope (Motic DM-143-FBGG) and identified to the lowest possible taxonomic level following Dill (2002) and Nguyen et al. (2013). The occurrence of each food item was calculated by the following equation: %*O*_*i*_ = 100 × *O*_*i*_/*N* (Hynes, 1950), where *O*_*i*_ is the number of fishes consuming prey *i* and *N* is the total number of fishes examined. The weight of each food item was calculated by the following equation: %*W*_*i*_ = 100 × *W*_*i*_/*W*_*total*_ (Hyslop, 1980), where, *W*_*i*_ is weight of prey *i*, *W*_*total*_ is total weight of all prey individuals. Biovolume of prey was calculated by the following equation: %*V*_*i*_ = (100 × *O*_*i*_ × *Wi*)/Σ(O_i_ × W_i_), where *V*_*i*_, *O*_*i*_ and *W*_*i*_ are the percentage of biovolume, occurrence and weight of prey *i* respectively. The diet composition was analysed against sex, size, site, and season to understand the interactions of these parameters to food acquisition (Natarajan & Jhingran, 1961; Hyslop, 1980; Clayton, 2017; Dinh et al., 2020b).

The modified graphical method of Costello (1990) was used to visualise the diet composition of *Ps. chrysospilos* (Amundsen, Gabler & Staldvik, 1996). Diet components that are highly abundant and essential appear at the upper right quadrant of the graph, while less abundant but essential components appear at the lower right quadrant of the graph; less essential components that are abundant or scarce appear on the left upper and lower quadrants respectively (Adámek, Andreji & Gallardo, 2007).

### Data analyses

PRIMER v.6.1.11 (Clarke & Gorley, 2006) with PERMANOVA+ v.1.0.1 add-on package (Anderson, Gorley & Clarke, 2008) was used to test if variation in diet can be attributed to the sex and size of the fish, or to site and season of catch (Baeck, Yoon & Park, 2013). If variations were detected, nonparametric tests would be applied to identify the driver for the differences (Dinh et al., 2017). The Mann-Whitney U test was used to test for changes in diet composition between factor pairs: sex (male and female individuals); size (immature and mature were determined basing on the *L*_*m*_ value as mentioned in the section on fish collection); and season (dry and wet). If diet composition varied for more than two factors, the Kruskal-Wallis H test was applied to identify the component driver.

T-test was applied to test the variations of RGL between fish sex, sizes, and season. One-way ANOVA was used to test the variation of RGL between sites. The effect of interactions between factors such as: gender × size; gender × season; gender × site; size × season; size × site; season × site; gender × size × season; gender × size × site; gender × season × site; size × season × site; and gender × size × season × site on RGL were quantified using General Linear Model. The significant level was *p* < 0.05 in all tests. To decrease the likelihood of Type I error of all test, the Benjamini–Hochberg procedure was applied (Benjamini & Hochberg, 1995; McDonald, 2014).

## Results

A total of 1,031 *Ps. chrysospilos* specimens were captured from four sites (508 females and 523 males) over a span of 1 year, from April 2020 to March 2021,. The highest quantity of fishes was collected from Dong Hai, Bac Lieu (303) while the lowest was from Dam Doi, Ca Mau (229). The number of *Ps*. *chrysospilos* recovered monthly from each site ranged from 10 to 42 fishes, including those with full and empty digestive tracts. The total length and weight of *Ps*. *chrysospilos* ranged 3.4–10.6 cm and 0.38–14.13 g, respectively. The most specimens of *Ps. chrysospilos*, at 116 individuals, was collected in July while the fewest specimens collected, at 64 specimens, was collected in October. Of these, the digestive tracts of 546 individuals were found with food, while the digestive tracts of the remaining 485 individuals were empty (see Table 1). Overall the ratio of fishes with empty *vs* full digestive tracts was approximating 1:1. Dong Hai, Bac Lieu was the site with the highest occurrence of individuals (164 individuals) with empty digestive tracts. The month with the least and most fishes with empty digestive tracts was July (56 individuals) and October (27 individuals) respectively.

**Table 1:** Distribution of *P. chrysospilos* specimens.

Year	Month	Duyen Hai, Tra Vinh				Tran De, Soc Trang				Dong Hai, Bac Lieu				Dam Doi, Ca Mau			
		Total specimens		Empty DT		Total specimens		Empty DT		Total specimens		Empty DT		Total specimens		Empty DT	
		M	F	M	F	M	F	M	F	M	F	M	F	M	F	M	F
2020	April	20	8	8	3	6	14	1	6	9	21	6	13	7	8	4	6
2020	May	10	7	4	2	12	12	6	5	12	8	5	4	11	6	5	4
2020	June	7	17	3	0	5	28	2	9	17	17	6	8	13	12	12	11
2020	July	14	7	6	2	19	9	4	4	12	18	10	13	16	15	7	10
2020	August	7	14	2	5	5	5	2	1	17	11	4	3	15	6	8	3
2020	September	13	6	6	2	13	4	6	4	26	16	22	12	11	9	3	1
2020	October	3	14	2	2	11	9	5	2	7	9	6	6	6	5	3	1
2020	November	6	11	3	2	13	7	7	3	14	5	8	3	11	6	4	3
2020	December	7	12	6	8	8	15	5	8	8	16	3	7	10	6	3	0
2021	January	9	9	7	6	10	16	4	7	5	15	2	2	11	6	2	1
2021	February	11	6	10	6	9	12	2	2	12	8	3	5	11	5	5	3
2021	March	13	8	8	4	9	9	6	6	7	13	7	6	15	8	5	3
**Total Specimens**		120	119	65	42	120	140	50	57	146	157	82	82	137	92	61	46

### Relative Gut Length (RGL)

The RGL values were significantly different between individuals of different sizes (t-test, *t* = −5.39, *n* = 1.031, df = 1.029, *p* < 0.001, CI_95%_ [−0.07 to −0.03]); mature individuals exhibited higher RGL values when compared to immature individuals (0.56 ± 0.006 and 0.51 ± 0.007), respectively, (see Table 2). However, the RGL values were not significantly different between males and females (*t* = 0.45, *n* = 1.031, df = 1.029, *p* = 0.15, CI_95%_ [−0.01 to 0.02]; see Table 2). When testing for temporal variations, RGL values were significantly different between seasons (*t* = −12.90, *n* = 1.031, df = 1.029, *p* < 0.001, CI_95%_ [−0.13 to −0.10]). The RGL value was higher in the wet season than the dry season (0.59 ± 0.01 and 0.47 ± 0.01, respectively, see Table 2). During the study duration, RGL values were the highest from July to November 2020 (around 0.61 ± 0.01–0.65 ± 0.016), while the lowest value was observed in January 2021 (0.44 ± 0.01) (One-way ANOVA, *F*_*2,11*_ = 28.22, *p* < 0.001, Tukey *Post Hoc* comparison analysis) (Table 2). When testing for spatial variations, RGL values were found to be significantly different between sites. The RGL value at Dam Doi, Ca Mau was the highest (0.57 ± 0.009, *F*_*2,3*_ = 3.52, *p* = 0.015), and the lowest was found in Duyen Hai, Tra Vinh and Tran De, Soc Trang (0.53 ± 0.01).

**Table 2:** The Relative Gut Length (RGL) values in *Periophthalmus chrysospilos*.

Category		Specimens	RGL (Mean ± SE)	t-test
Sex	Female	508	0.55 ± 0.006^a^	*t* = 0.45, *p* = 0.15
	Male	523	0.54 ± 0.007^a^	
Season	Dry	408	0.47 ± 0.006^a^	*t* = −12.90, *p* < 0.01
	Wet	623	0.59 ± 0.006^b^	
Size	Immature	377	0.51 ± 0.007^a^	*t* = −5.39, *p* < 0.01
	Mature	654	0.56 ± 0.006^b^	
			RGL (Mean ± SE)	One way-ANOVA
Year, Month	2020 April	93	0.47 ± 0.01^abc^	*F* = 28.22, *p* < 0.01
	2020 May	78	0.45 ± 0.008^ab^	
	2020 June	116	0.52 ± 0.012^cd^	
	2020 July	110	0.61 ± 0.01^e^	
	2020 August	80	0.65 ± 0.016^e^	
	2020 September	98	0.61 ± 0.018^e^	
	2020 October	64	0.64 ± 0.02^e^	
	2020 November	73	0.63 ± 0.019^e^	
	2020 December	82	0.50 ± 0.017^bcd^	
	2021 January	81	0.44 ± 0.01^a^	
	2021 February	74	0.48 ± 0.02^abcd^	
	2021 March	82	0.54 ± 0.02^de^	
Site	Duyen Hai, Tra Vinh	239	0.53 ± 0.009^a^	*F* = 3.52, *p* = 0.015
	Tran De, Soc Trang	260	0.53 ± 0.01^a^	
	Dong Hai, Bac Lieu	303	0.55 ± 0.009^ab^	
	Dam Doi, Ca Mau	229	0.57 ± 0.009^b^	

**Note:**

Different letters (a, b, c, d and e) showed variation of RGL in each category.

Although the RGL of *Ps. chrysospilos* varied ontogenetically, all values were *RGL* < 1, indicating that this species is primarily carnivorous (Al-Hussaini, 1947). The interaction between factors, as assessed using GLM, such as gender × size (*F*_*2,1*_ = 1.48, *p* = 0.23); gender × season (*F*_*2,1*_ = 0.78, *p* = 0.38); gender x site (*F*_*2,1*_ = 0.92, *p* = 0.43); size × season (*F*_*2,1*_ = 0.24, *p* = 0.63); size × site (*F*_*2,1*_ = 1.39, *p* = 0.25); gender × size × season (*F*_*2,2*_ = 1.94, *p* = 0.16); gen × size × site (*F*_*2,2*_ = 2.31, *p* = 0.08); gender × season × site (*F*_*2,2*_ = 0.48, *p* = 0.70); size × season × site (*F*_*2,2*_ = 0.89, *p* = 0.45); gender × size × season × site (*F*_*2,3*_ = 0.33, *p* = 0.81), did not exhibit significant impact to the RGL with the exception of interaction season × site (*F*_*2,1*_ = 3.93, *p* = 0.008).

### Diet composition

**Figure 2:**
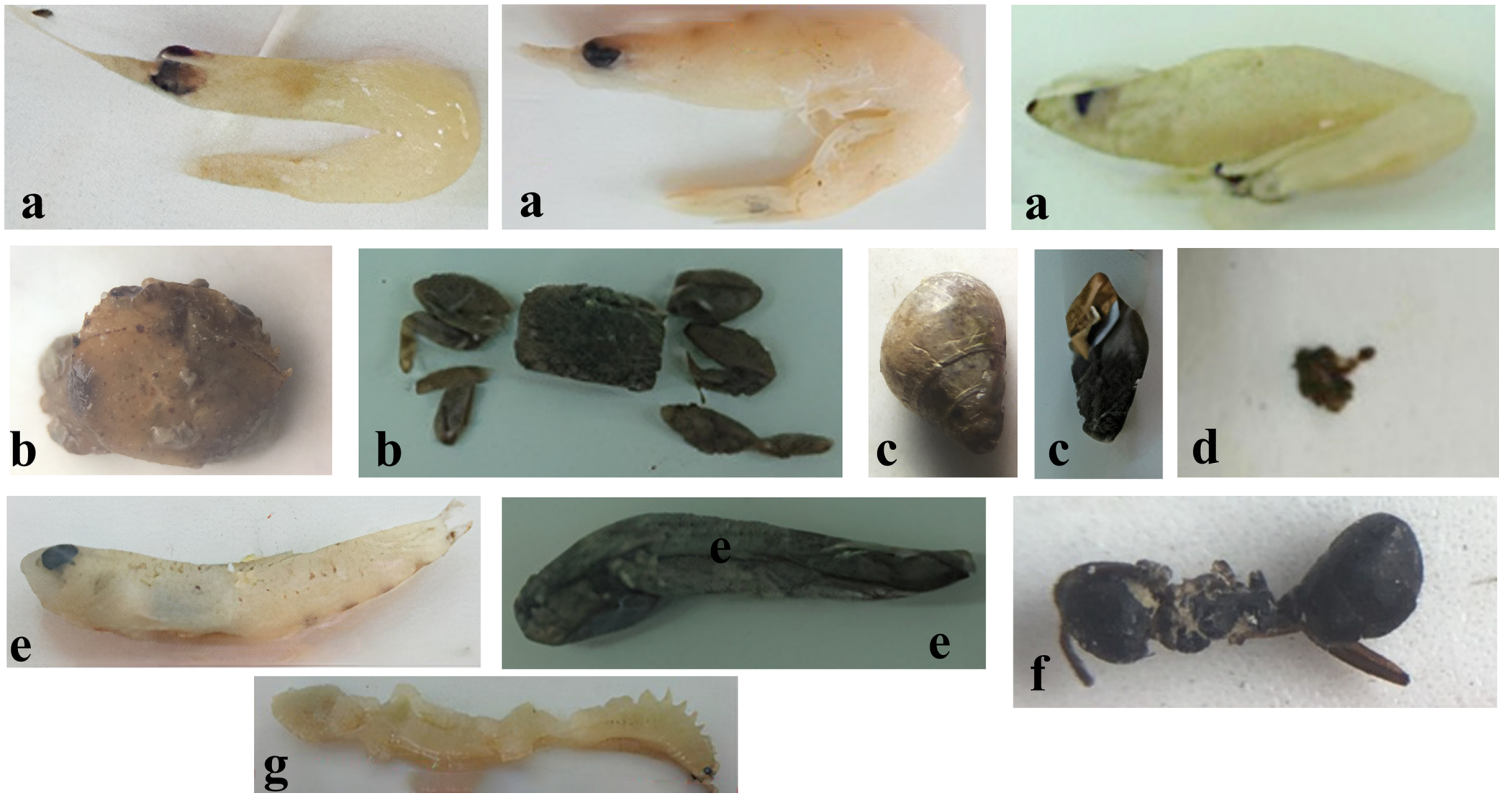
The food composition of *Periophthalmus chrysospilos*. (A) *Acetes* spp., (B) *Uca* spp., (C) Mollusca, (D) Detritus, (E) Actinopterygii, (F) *Dolichoderus* sp., and (G) Polychaeta.

Analyses of the digestive tracts of 546 individuals of *Ps*. *chrysospilos* revealed clear patterns of food preferences (see Table 3 and Fig. 2). The three most-dominant food items from the pooled data of all individuals were, in descending order of percentage biovolume—*Acetes* spp. (small shrimps, 26.8%), *Uca* spp. (fiddler crabs, 21.6%) and *Dolichoderus* sp. (ant, 19.7%). Other food items present, by percentage biovolume, were Mollusca (molluscs, 16.7%), Polychaeta (bristleworms, 3.4%) and Actinopterygii (ray-finned fishes, 0.7%). Although relatively high in biovolume (11.2%) and occurrence (26.0%), detritus was low in weight (6.6% of total weight).

**Table 3:** Recovered food items from digestive tracts (DT) of *Periophthalmus chrysospilos* by percentage occurrence, percentage weight, and percentage biovolume.

Food Item	% Biovolume	% Weight	% Occurrence
*Acetes* spp.	26.8	21.5	19.2
*Uca* spp.	21.6	24.3	13.7
*Dolichoderus* sp.	19.7	16.2	18.8
Detritus	11.2	6.6	26.0
Mollusca	16.7	18.6	13.8
Polychaeta	3.4	9.4	5.6
Actinopterygii	0.7	3.4	2.9

**Figure 3:**
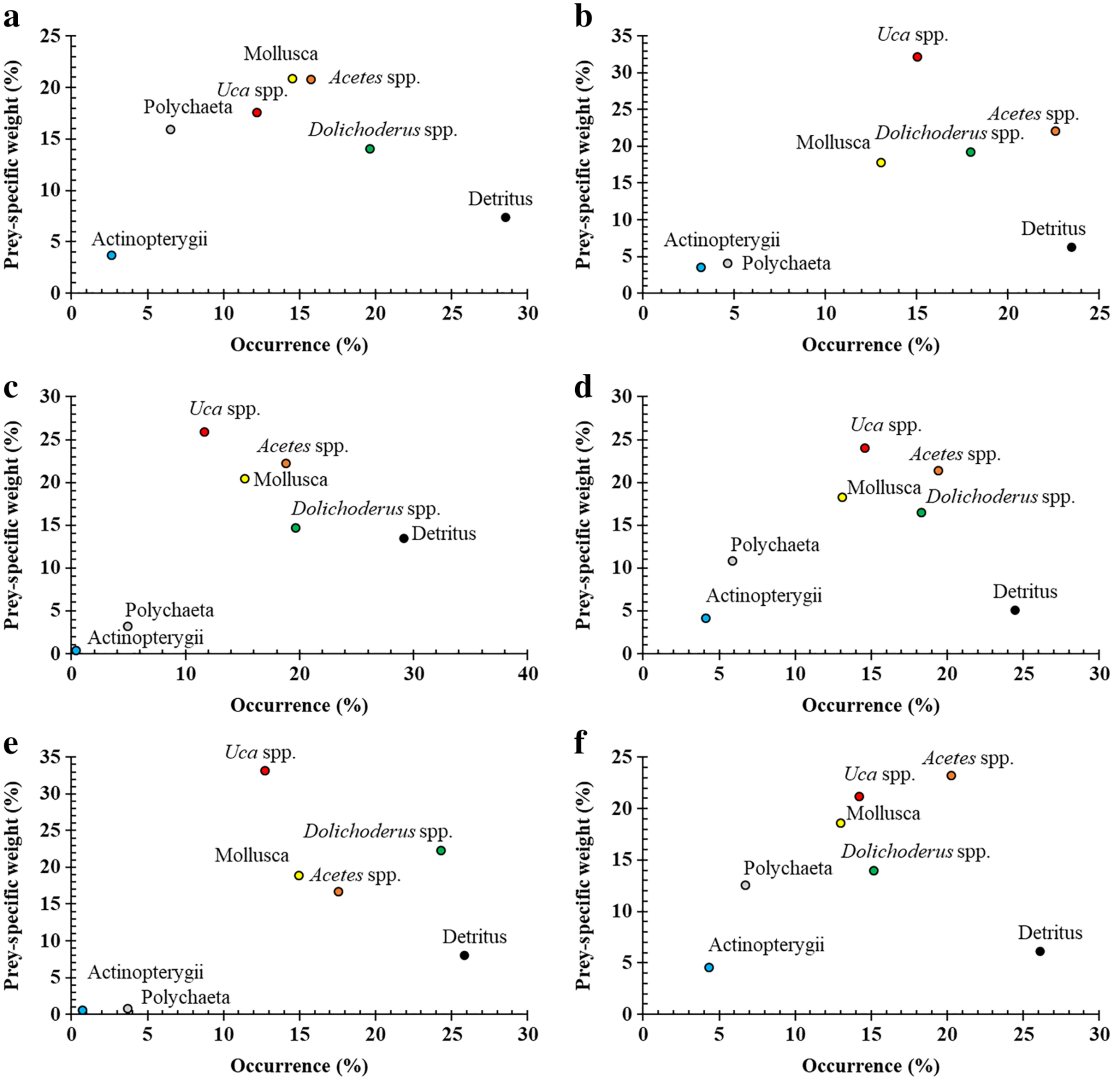
Modified Costello Graphs represent the feeding strategy of *Periophthalmus chrysospilos* based on the relationship between the percentage weight and percentage occurrence of food items between male and female individuals. (A) male, *n* = 523; (B): female, *n* = 508); immature and mature individuals ((C): immature, *n* = 377; (D): mature, *n* = 654); and dry and wet seasons ((E): dry season, *n* = 408; (F): wet season, *n* = 623.

The variation of occurrences, weight and biovolume of food items when compared to the sex and size of the fishes, as well as the site and season of catches, are illustrated in Table 4. The diet composition of *Ps. chrysospilos* did not vary under season and size parameters (PERMANOVA, *df* = 1, *Pseudo-F*_*season*_ = 1.64, *p* = 0.16; *df* = 1, *Pseudo-F*_*fish size*_ = 0.54, *p* = 0.75) but did vary under the sex and site parameters (*df* = 1, *Pseudo-F*_*gender*_ = 3.86, *p*_*gender*_ = 0.003; *df* = 1, *Pseudo-F*_*sampling sites*_ = 3.80, *p*_*sampling sites*_ = 0.001). The only diet component that drove the variance between males and females was ‘*Uca* spp.’ (Mann-Whitney U, *df* = 1, *U* = -1.93, *p* = 0.05), with females consuming more of these crabs than their male counterparts (see Fig. 3). The variation observed between sites were driven by five of the diet components: Mollusca, *Uca* spp., *Dolichoderus* sp. and detritus (Kruskal Wallis H test, *df* = 3, *χ*^*2*^_*Mollusca*_ = 16.98, *p*_*Mollusca*_ = 0.001; *df* = 3, *χ*^*2*^_*Uca*_ _spp._ = 8.15, *p*_*Uca*_ _spp._ = 0.04; *df* = 3, *χ*^*2*^_*Dolichoderus*_ = 12.01, *p*_*Dolichoderus*_ = 0.007; *df* = 3, *χ*^*2*^_*detritu*_ = 65.17, *p*_*detritus*_ < 0.001). The three other food items, *Acetes* spp., Polychaeta and Actinopterygii, were not significant contributors for the variation observed between sites (*df* = 3, *χ*^*2*^_*Acetes*_ = 2.39, *p*_*Polychaeta*_ = 0.50; *df* = 3, *χ*^*2*^_*Polychaeta*_ = 7.51, *p*_*Polychaeta*_ = 0.06; *df* = 3, *χ*^*2*^_*Actinopterygii*_ = 3.63, *p*_*Polychaeta*_ = 0.30).

**Table 4:** Percentage occurrence, percentage weight, and percentage biovolume of recovered food items within the digestive tracts of *Periophthamus chrysospilos* according to sex, season, and site (DHTV: Duyen Hai, Tra Vinh; TDST: Tran De, Soc Trang; DHBL: Dong Hai, Bac Lieu; DDCM: Dam Doi, Ca Mau.

	Parameter	*Acetes* spp.	Actinopterygii	Mollusca	*Uca* spp.	Polychaeta	*Dolichoderus* sp.	Detritus	PERMANOVA	
									*Pseudo-F*	*p*
% Occurrence	Female	22.6	3.2	13.0	15.1	4.6	18.0	23.5	3.86	0.003
	Male	15.8	2.7	14.6	12.2	6.6	19.6	28.6		
% Weight	Female	22.1	3.2	16.7	30.2	3.8	18.0	5.9		
	Male	20.7	3.7	20.8	17.5	15.9	14.0	7.4		
% Biovolume	Female	30.0	0.6	13.1	27.4	1.1	19.5	8.4		
	Male	22.7	0.7	21.0	14.8	7.2	19.0	14.6		
Mann-Whitney U test	*U*	−1.66	−1.32	−0.40	−1.93	−0.15	−0.37	−1.02		
	*p*	0.10	0.19	0.69	0.05	0.88	0.71	0.31		
% Occurrence	Dry	17.6	0.8	15.0	12.7	3.8	24.3	25.8	1.64	0.162
	Wet	20.3	4.4	13.0	14.3	6.8	15.2	26.1		
% Weight	Dry	16.6	0.5	18.9	33.1	0.7	22.2	8.0		
	Wet	23.2	4.5	18.6	21.1	12.6	13.9	6.1		
% Biovolume	Dry	16.7	0.0	16.2	24.1	0.2	31.0	11.9		
	Wet	31.6	1.3	16.2	20.2	5.7	14.2	10.7		
% Occurrence	Immature	18.8	0.5	15.3	11.7	4.9	19.7	29.2	0.54	0.747
	Mature	19.4	4.2	13.1	14.6	5.9	18.3	24.5		
% Weight	Immature	22.2	0.3	20.4	25.9	3.2	14.6	13.5		
	Mature	21.3	4.1	18.3	24.0	10.8	16.5	5.1		
% Biovolume	Immature	24.2	0.0	18.0	17.5	0.9	16.7	22.7		
	Mature	27.4	1.1	15.8	23.2	4.2	20.0	8.3		
% Occurrence	DHTV	20.4	3.1	15.4	8.0	6.2	26.5	20.4	3.80	0.001
	TDST	17.4	5.1	22.5	18.0	9.6	17.4	10.1		
	DHBL	18.9	2.4	10.1	12.4	3.6	18.9	33.7		
	DDCM	20.4	1.2	7.0	15.7	2.9	12.8	40.1		
% Weight	DHTV	19.4	3.3	16.2	16.0	0.7	36.0	8.5		
	TDST	18.4	3.6	25.6	18.7	19.7	12.4	1.6		
	DHBL	25.5	2.8	16.6	24.8	3.8	18.4	8.2		
	DDCM	23.1	4.1	10.5	40.1	4.6	5.3	12.3		
% Biovolume	DHTV	20.6	0.5	13.0	6.7	0.2	49.8	9.1		
	TDST	19.2	1.1	34.4	20.1	11.3	13.0	1.0		
	DHBL	30.2	0.4	10.5	19.2	0.8	21.7	17.2		
	DDCM	26.9	0.3	4.2	35.9	0.8	3.8	28.1		
*Kruskal Wallis H test*	*χ* ^*2*^	2.39	3.63	16.98	8.15	7.51	12.01	65.17		
	*p*	0.50	0.30	0.00	0.04	0.06	0.01	<0.01		

**Figure 4:**
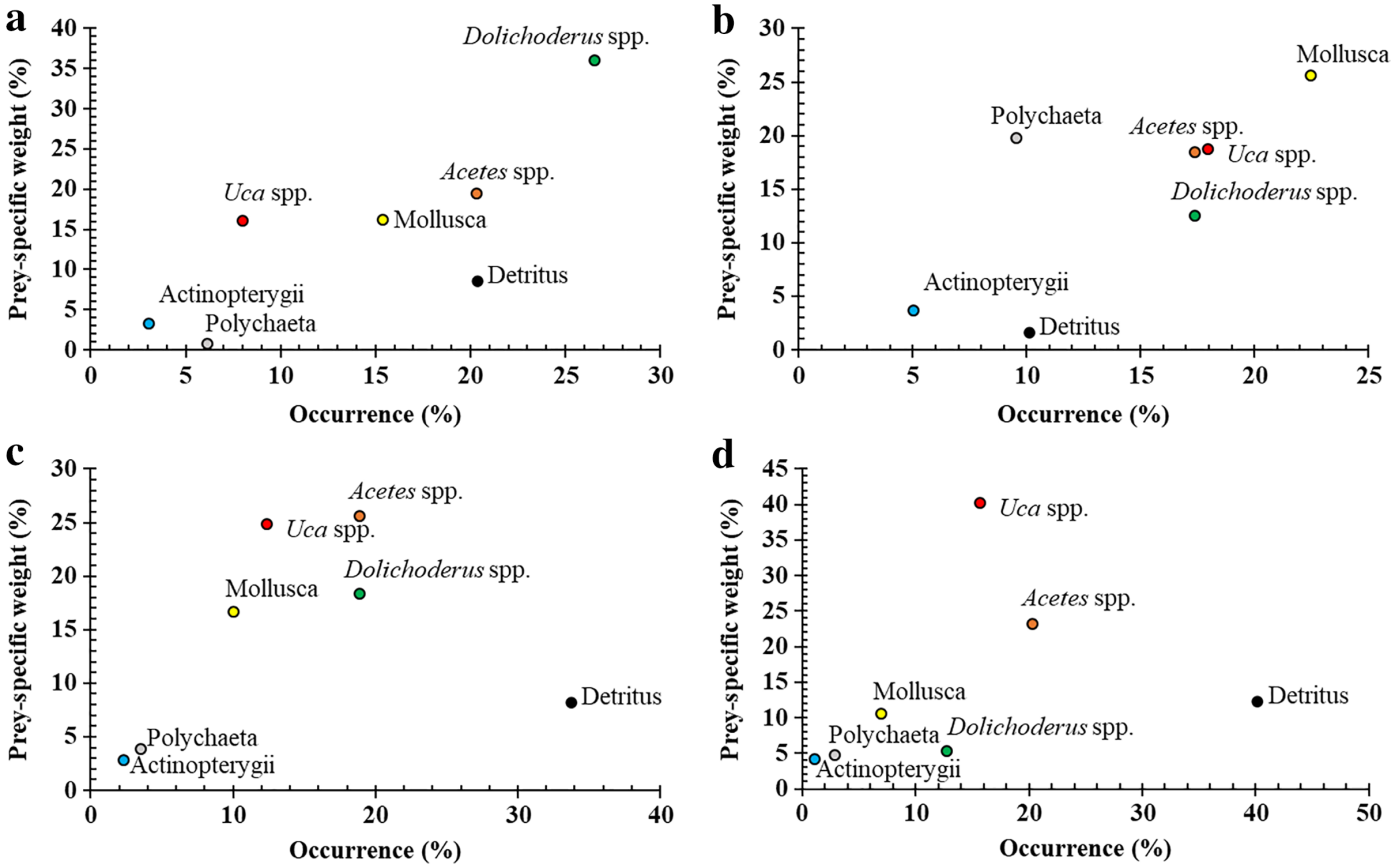
Modified Costello Graphs represent the feeding strategy of *Periophthalmus chrysospilos* based on the percentage weight and percentage occurrence of food items at four sampling sites. (A) Duyen Hai, Tra Vinh, *n* = 239; (B) Tran De, Soc Trang, *n* = 260; (C) Dong Hai, Bac Lieu, *n* = 303; (D) Dam Doi, Ca Mau, *n* = 229.

Visualisations on the Costello graph (see Fig. 3) revealed *Acetes* spp. and *Uca* spp. to be the most significant prey items for *Ps*. *chrysospilos*, followed by *Dolichoderus* sp. and Mollusca with equal significance. Detritus, also high in occurrence, was not considered significant based on the position of this diet component within the lower right quadrant of the Costello graph. Both Polychaeta and Actinopterygii were considered less important prey items reflected by their positions within the lower left quadrant of the Costello graph. The diet composition of individuals of *Ps*. *chrysospilos* at the four sites (see Fig. 4) were significantly different. At DHTV, *Dolichoderus* sp. was the most important food item; at TDST, Mollusca was the item with the most significant contribution. At DHBL, *Acetes* spp. accounted for 30.2% biovolume, and was also by far, the most important prey item to *Ps. chrysospilos* here. At DDCM, *Uca* spp. was the main prey item and accounted for 35.9% of the biovolume of the mudskippers occurring at this site.

## Discussion

*Periophthalmus chrysospilos* is an opportunistic carnivore based on its relative gut length and the contents within the digestive tracts of specimens examined. The RGL of this species fluctuated between values of 0.44 and 0.65, and falls within the range of a carnivorous fish with moderate feeding intensity (Al-Hussaini, 1947). These values corroborate those previously obtained for other *Periophthalmus* species (Clayton, 1993). An ontogenetic shift in the relative gut length was observed in this species, with larger specimens having longer digestive tracts. Seasonal variations in the RGL values could be attributed to the abundant available nutrient for rapid fish development during the wet season (July to November) (Hortle, 2009). The highest RGL values were disproportionately recorded from specimens collected at DDCM, an area with the highest flora diversity amongst sites. The variation in RGL values between sites was also reported in other confamilial taxa—*Periophthalmodon septemradiatus* (Dinh, Tran & Nguyen, 2018) and *Butis koilomatodon* (Nguyen, Lam & Dinh, 2020)—from the Mekong Delta. The varied diet composition of this amphibious fish species reflects the habitat in which it resides and the interactions between marine and terrestrial biota within the intertidal areas. The dominant diet component of this species is *Acetes* spp, small marine shrimps that are not tolerant of emersion out of water. The second-most dominant component of its diet is the fiddler crabs, *Uca* spp, that are active on exposed sand or mudflats during the ebb tide. Next in dominance by diet composition is *Dolichoderus* sp., terrestrial ants that are found at or near mangrove forests.

Within the Mekong Delta, *Acetes* spp., *Uca* spp*., Dolichoderus* sp. and many molluscs species are commonly available (Cuc & Van Mele, 1999; Dinh et al., 2020b). Their availability at the sites determines their dominance in the diet composition of this mudskipper species. For example, *Dolichoderus* sp. was the most important prey item for specimens collected at DHTV, while *Acetes* spp. was the main food item for specimens from DHBL. Unsurprisingly, the highest diet components of *Ps*. *chrysospilos* from elsewhere differ slightly to those from our study; specimens of this species from southern Sumatra predominantly consumed *Uca* sp. and fish eggs (Ridho, Patriono & Solikha, 2019). Similar to our study, detritus accounted for a significant proportion of the diet composition. However, as with our study, polychaete worms and small fishes were rarely encountered as diet components of specimens from Sumatra, Indonesia (Ridho, Patriono & Solikha, 2019) and Tanjung Piai, Peninsular Malaysia (Hui et al., 2019). The tolerance to a wide diversity of food items suggests the adaptability of this species to constant environmental changes within the intertidal habitats.

The diet composition of *Ps*. *chrysospilos* was not affected by season and fish size but differed according to sex and sites. Females consumed more *Uca* spp. than males. Gender-associated feeding modes was also observed in the congener *Ps*. *barbarus* (Linnaeus, 1766)—more cyanobacteria, *Coscinodiscus* spp. and *Sesarma* spp. were found in the digestive tracts of males than females (Udo, 2002b). This differential preference in food intake was also observed in a closely-related genus *Periophthalmodon* (hereafter ‘*Pn’.)*, in which the males of *Pn. schlosseri* typically consumed *Uca* spp. while females exhibited preference for the ricefish *Oryzias* sp. (Zulkifli et al., 2012). The site, and by extension the food availability therein, was a driver for the differences in the main food item ingested by *Ps*. *chrysospilos* —*Dolichoderus* sp., Mollusca, *Acetes* spp. and *Uca* spp. were the principal prey item for specimens collected from DHTV, TDST, DHBL and DDCM respectively. Similarly, the diet of congener *Ps*. *argentilineatus* from Zanzibar consisted predominantly of amphipods and copepods, whereas in mainland Tanzania, Polychaeta was the main food source (Kruitwagen et al., 2007).

There appear to be no changes in food preferences between *Ps*. *chrysospilos* of size ranges from 3.4 cm to 10.6 cm in this study, an observation contrary to mudskippers of other species. *Periopththalmus argentilineatus* collected from Iriomote Island (Japan) showed a significant shift in dietary preference from Polychaetae in smaller individuals to *Uca* spp. for larger individuals (Nanjo, Kohno & Sano, 2008). The contribution of *Uca* spp. is more significant in larger and mature specimens of *Pn. schlosseri* (Zulkifli et al., 2012) and *Parapocryptes serperaster* (Dinh et al., 2017); the dietary composition of smaller individuals of the latter species consisted mostly of *Dolichoderus* sp. (Dinh et al., 2017). The dietary composition of *Ps*. *chrysospilos* is independent of seasons, unlike its congener *Ps. barbarus* (observed in the Niger Delta) that exhibited a shift to the significantly higher consumption level of three food items, namely unidentified crab parts, *Penaeus* sp, as well as annelid and nematode worms in the wet season compared to the dry season (Chukwu & Deekae, 2013). Analyses of digestive tracts also indicate that detritus and organic matter are ingested incidentally by this species. Despite the increased availability of these two items during the wet season (Nedeco, 1993), their composition within the diet of *Ps*. *chrysospilos* remained similar between seasons The dominance of detritus to dietary composition in *Ps. barbarus* (from Nigeria) however, persisted only during the wet season but changed to algae-dominant in the wet season (Udo, 2002a).

In conclusion, *Periophthalmus chrysospilos* is demonstrated to be an opportunistic mesopredator, and a carnivore with moderate feeding intensity, as evidenced by the RGL value (<1) and an equal ratio of fishes with empty and full digestive tracts. The food composition of *Ps. chrysospilos* consisted of seven types of food items: *Acetes spp*., *Uca spp*., *Dolichoderus sp*., Mollusca, Polychaeta, Actinopterygii, and detritus. The principal food item of this mudskipper species varied depending on site and food availability, thus indicating its adaptability in pursuing prey items within the limits of littoral zone. Exploited organisms range from marine shrimps to intertidal crabs and terrestrial ants, signalling the importance of this amphibious species as a conduit of terrestrial-marine connectivity within littoral landscapes.

## Supplemental Information

10.7717/peerj.12582/supp-1 Supplemental Information 1 Raw data.

## Additional Information and Declarations

### Data Availability

The following information was supplied regarding data availability: The raw data is available in the Supplemental File.
